# Δ113p53/Δ133p53: survival and integrity

**DOI:** 10.18632/oncotarget.4371

**Published:** 2015-06-08

**Authors:** Jun Chen, Jinrong Peng

**Affiliations:** College of Animal Sciences, Zhejiang University, Hangzhou, China

In 2005, two reports, one on analysis of 5′-RACE PCR products of p53 transcripts in human normal tissues [1] and the other on analysis of a zebrafish genetic mutant *def^hi429^* [2], described the discovery of p53 isoforms. Since then it has raised tremendous interest in the cancer research community to unravel the function and regulation of these isoforms due to the fact that p53 (TP53) is the most important tumor suppressor. During last ten years, main focus has been put on studying one unique type of *p53* isoforms, namely the zebrafish *∆113p53* and its human counterpart *∆133p53*, both are transcribed by an alternative *p53* promoter in intron 4 and encodes N-terminal truncated forms of p53 [3]. In summary, these studies demonstrated that *∆113p53/∆133p53* are p53 target genes and function to antagonize p53's apoptotic function or to inhibit cell replicative senescence [1, 3, 4, 5]. Recently, four zebrafish p53 isoforms, namely TA2/3/4/5p53 were identified. These isoforms are derived from a naturally occurring 4 bp genomic deletion in the intron 1 of the *ß113p53* gene (part of the intron 4 of the *p53* gene) [6]. All of these new isoforms also promote embryo survival by repressing apoptosis upon γ-irradiation [6].

DNA double strand breaks (DSB) are the most deleterious DNA lesions that a cell can encounter. To combat these detrimental insults, three pathways have evolved for the repair of DSB: Homologous Recombination (HR), Non-Homologous End Joining (NHEJ) and Single-Strand Annealing (SSA). It is well established that full-length p53 represses DNA DSB repair by either preventing repair complex formation through interacting with several essential HR-related proteins such as RAD51 and RPA, or transcriptionally suppressing the expression of DNA DSB repair genes e.g. RAD51, RECQ4 and WRN [7]. This is consistent with the observation that p53 protein is rapidly accumulated to a high level in response to DNA DSB stress at early stage. However, activated p53 also activates the expression of MDM2, an E3 ligase that mediates the degradation of p53 at the later stage. Therefore, it is of great interest to understand the role of the activated p53 signal pathway in response to DNA DSB stress at the later stage.

We showed previously that, in response to DNA damage, ∆113p53/∆133p53 is strongly induced to antagonizes p53 apoptotic activity specifically [3]. We asked whether p53 isoforms are involved in the DNA DSB repair. Our study, recently published in Cell Research, demonstrates that in response to DNA DSB stress, ß113p53/ß133p53 not only inhibits apoptosis, but also minimizes genetic insults by promoting all three DNA DSB repair pathways: HR, NHEJ and SSA, independently of p53 [8]. Firstly, we found that upon γ-irradiation, the accumulation of ß113p53/ß133p53 protein was different from that of full-length p53. p53 peaked as early as 4 hours post irradiation (hpi) and gradually decreased. ß113p53/ß133p53 protein reached its highest level at 24 hpi, at the same time p53 decreased to its basal level. Interestingly, induction of apoptosis was correlated positively with the level of p53 and negatively with the level of ß113p53, whereas DNA damage repair was corresponding to the highest level of ß113p53. Then, using artificial reporter systems, we revealed that ß113p53/ß133p53 promotes all three DNA DSB repair pathways in a p53-independent manner. *In vivo* analysis showed that the knockdown of ß113p53/ß133p53 reduces DNA DSB repair foci formation and increases DNA damage extents after γ-irradiation. The high level of DNA damage results cell growth arrest at G2 phase which finally leads to cell senescence at the later stage. The significance of ß113p53 induction upon γ-irradiation was further demonstrated in the zebrafish *ß113p53* knockout mutant by deleting one p53-responsive element in its promoter. The *ß113p53* knockout mutant embryos are more sensitive to γ-irradiation due to accumulating higher level of DNA damage extents and apoptotic cells. The results demonstrated that both of functions of ß113p53/ß133p53 in antagonizing apoptosis and promoting DNA DSB repair are important for its pro-survival characters. Finally, we demonstrated that ß113p53/ß133p53 promotes DNA DSB repair through up-regulating the expression of the DNA DSB repair genes *rad51*, *ligaseIV* (*lig4*) and *rad52*, which is independent of full-length p53. Promoter analysis showed that ß113p53 binds to a novel type of p53 RE in the promoters of the three genes.

Hence, we revealed that p53 coordinates with its isoform ß113p53/ß133p53 to protect genomic stability upon ionizing irradiation: at the early stage after irradiation, full-length p53 is quickly accumulated to a high level in cells harboring severe DNA damage to inhibit DNA DSB repair and to guide such cells to undergo apoptosis, whereas p53 protein is accumulated to a relative low level in cells with less and fixable DNA damage, thus to activate the transcription of its target genes including *mdm2* and *ß113p53/ß133p53*. The expression of mdm2 promotes p53 protein degradation, but not ß113p53/ß133p53 because ß113p53/ß133p53 lacks the mdm2-interacting motif. Therefore, at the later stage, ß113p53/ß133p53 protein can accumulate to higher levels in cells with less DNA damage as p53 protein decreases to the basal level. The accumulation of ß113p53 not only protects cells from death by its anti-apoptotic function but also ensures genetic stability by promoting DNA DSB repair.
